# A novel saline-based occlusion tool allows for dye-less cryoballoon-based pulmonary vein isolation and fluoroscopy reduction

**DOI:** 10.3389/fcvm.2023.1156500

**Published:** 2023-03-24

**Authors:** Laura Rottner, Julius Obergassel, Katrin Borof, Ilaria My, Fabian Moser, Marc Lemoine, Jan-Per Wenzel, Paulus Kirchhof, Feifan Ouyang, Bruno Reissmann, Andreas Metzner, Andreas Rillig

**Affiliations:** ^1^ Department of Cardiology, University Heart and Vascular Center Hamburg, Hamburg, Germany; ^2^ Institute of Cardiovascular Sciences, University of Birmingham, Birmingham, UK; ^3^ German Center for Cardiovascular Research (DZHK), Partner Site Hamburg/Kiel/Lübeck, Hamburg, Germany

**Keywords:** atrial fibrillation, dielectric imaging, occlusion tool, saline injection, dye-less cryoballoon-based ablation, fluoroscopy reduction fluoroscopy reduction with saline injection atrial fibrillation, fluoroscopy reduction

## Abstract

**Background:**

Cryoballoon (CB)- based pulmonary vein isolation (PVI) remains guided by fluoroscopy and dye. The novel saline injection-based occlusion tool allows for pulmonary vein (PV)-occlusion assessment without the need for dye injection.

**Aim:**

To compare KODEX-EPD guided CB-PVI using the novel saline injection-based workflow with conventional cryoablation for acute efficacy, fluoroscopy exposure and dye volume.

**Methods:**

Consecutive atrial fibrillation (AF)- patients undergoing CB-PVI in conjunction with KODEX-EPD (Cryo^EPD^ group) were analyzed. Patients undergoing conventional CB-PVI (Cryo group) in the same time period acted as controls.

**Results:**

One hundred forty patients [91/140 (65%) persistent AF] were studied. Seventy patients underwent Cryo^EPD^ procedures [64 ± 13 years, 21 (30%) female] and seventy patients underwent Cryo procedures [68 ± 10 years, 27 (39%) female].

A total of 560 PVs were identified and successfully isolated. Mean procedure time was 66 ± 15 min for the Cryo^EPD^ group, and 65 ± 19 min for the Cryo group (*p* = 0.3). Fluoroscopy time (Cryo^EPD^ 6 ± 4 min; Cryo 13 ± 6 min, *p* < 0.001) and dose area product (Cryo^EPD^ 193 [111; 297] cGycm^2^; Cryo 381 [268; 614] cGycm^2^, *p* < 0.001) were lower in patients undergoing Cryo^EPD^ compared with Cryo procedures. No dye was needed in the Cryo^EPD^ group while 53 ± 18 ml dye per patient were administered for the Cryo group (*p* < 0.001). The overall complication rate was comparable between both groups (*p* = 0.5).

**Conclusion:**

KODEX-EPD guided AF-ablation enables dye-free CB-based PVI with reduced fluoroscopy exposure when compared to conventional CB-ablation, without differences in acute procedural outcomes or procedure duration.

## Graphical Abstract

Key results from a direct comparison of cryoballoon-based pulmonary vein isolation using a novel saline-based occlusion tool (Cryo^EPD^) and the conventional cryoablation approach (Cryo).

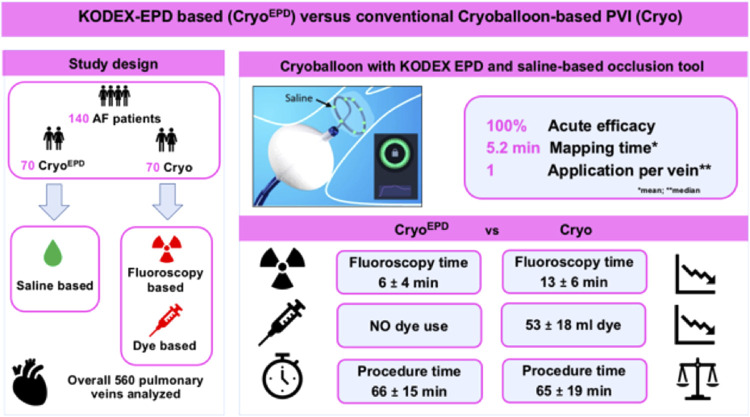

## Introduction

Catheter ablation is an established treatment option in symptomatic patients suffering from atrial fibrillation (AF) (1). In addition, early rhythm control has been shown to be favorable in the reduction of cardiovascular events in the main cohort of the EAST-AFNET 4 trial and in subgroup analyses (2, 3). Therefore, the most effective rhythm control strategy, which is catheter ablation is gaining more and more importance (4). Within this context, cryoballoon (CB) based AF ablation plays a major role as a first-line ablation tool because of its safety (5), high reproducibility (6) and efficacy (7, 8). A current limitation of CB ablation, however, is the need for fluoroscopy and dye injection throughout the procedure.

Recently, KODEX-EPD (EPD Solutions, Philips, Netherlands), a novel high-resolution cardiac imaging system was introduced, providing an occlusion tool using dielectric sensing to assess PV occlusion non-fluoroscopically (9, 10). Previous studies have shown a trend towards fluoroscopy reduction when using KODEX-EPD and its occlusion tool for CB—based PVI when using the dye-based approach (11, 12). A single smaller study on saline based CB ablation has shown promising results regarding feasibility, however comparison to conventional CB approaches have not been reported yet.

This study reports on the feasibility, acute efficacy and safety of CB-based PVI in combination with KODEX-EPD and the saline injection workflow for PV-occlusion assessment, when compared to conventional CB-based PVI in clinical routine in a large electrophysiological (EP) center.

## Methods

### Study population

Consecutive patients suffering from AF undergoing CB-based catheter ablation in conjunction with KODEX-EPD and the novel saline injection workflow (Cryo^EPD^ group) were retrospectively analyzed. Consecutive patients undergoing conventional CB-PVI (Cryo group) during the same time period acted as controls.

Exclusion criteria were prior ablation procedures, a left atrial (LA) diameter >55 mm and severe valve disease.

All patients gave confirmed consent. Data analysis and handling was performed in accordance to the Declaration of Helsinki (Local ethical code WF-021/20).

#### The KODEX-EPD occlusion tool

**Figure 1:**
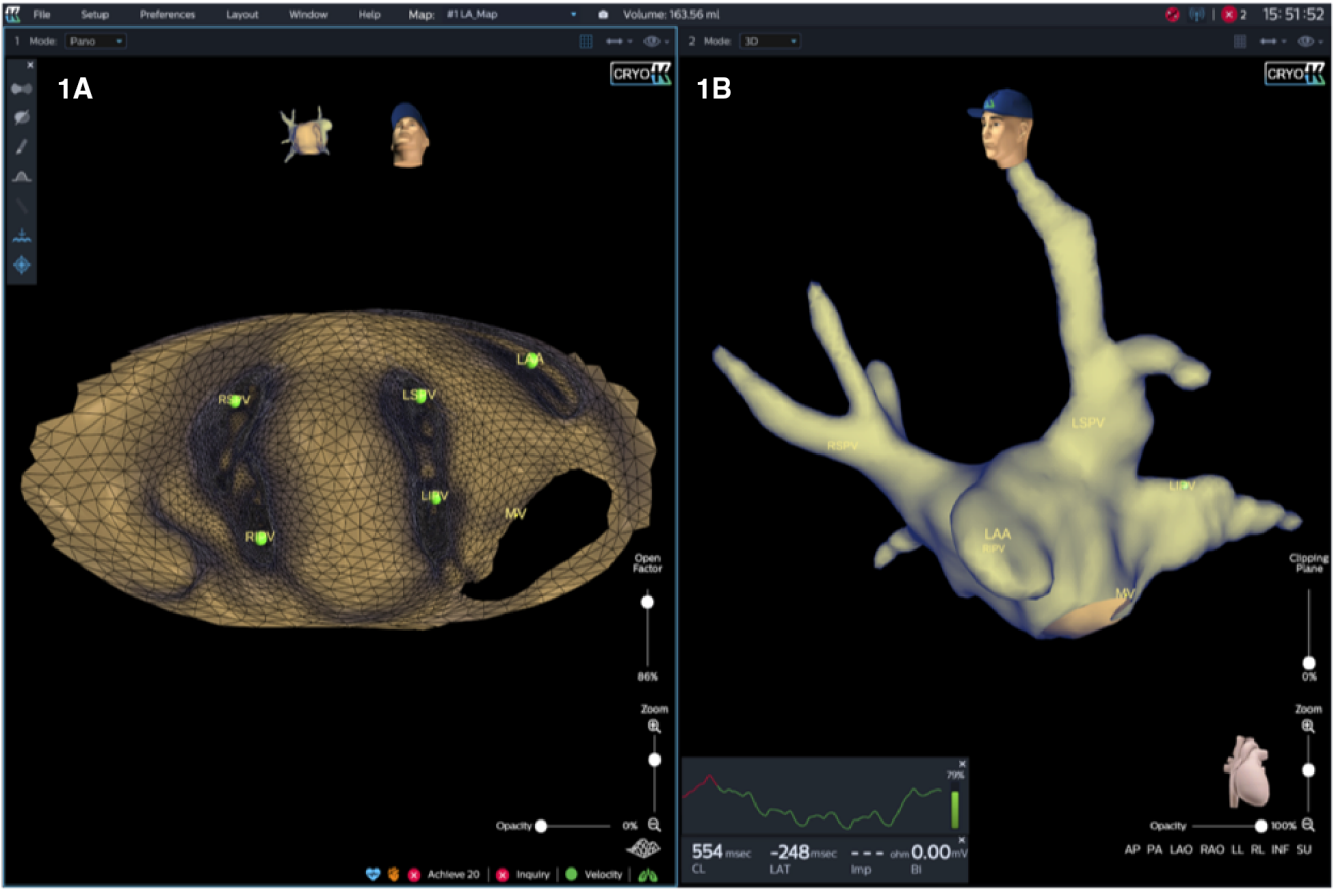
KODEX-EPD offers two options for the operator to visualize the cardiac anatomy. First, a 3D surface image, which provides a conventional shell of the heart chamber and may be rotated freely (**B**). Second, in an innovative approach, the heart may be opened virtually across the 3D surface and this flattened 3D panoramic view (“PANO” view) offers a visualization of the endocardial surface (**A**).

A novel mapping system (KODEX-EPD, EPD Solutions, Philips, Netherlands) based on dielectric properties was used for electroanatomic imaging, catheter navigation and assessment of PV-occlusion (Figure 1).

KODEX-EPD has two tools for sensing PV-occlusion: First, the baseline tool, and second, the injection tool. In our cohort, the injection-based workflow*,* which has been lately added in the R1.4.7 software version, was used in all patients. A major difference of the injection workflow is that it features a simplified workflow, which allows for a higher accuracy compared to the baseline workflow, in particular for detection of smaller leaks (9). Prior to injecting of a solution, the mapping catheter senses the baseline electric field around the electrodes and establishes a reference measurement for each electrode. The injection of contrast or saline, displaces the blood and the catheter electrodes sense the transient local changes in the electric field. Both fluids induce a characteristic immediate transient local change in conductivity sensed by the corresponding electrodes of the circular mapping catheter (Achieve, Medtronic Inc, Minneapolis, United States) and reflect the state of PV occlusion.

**Figure 2:**
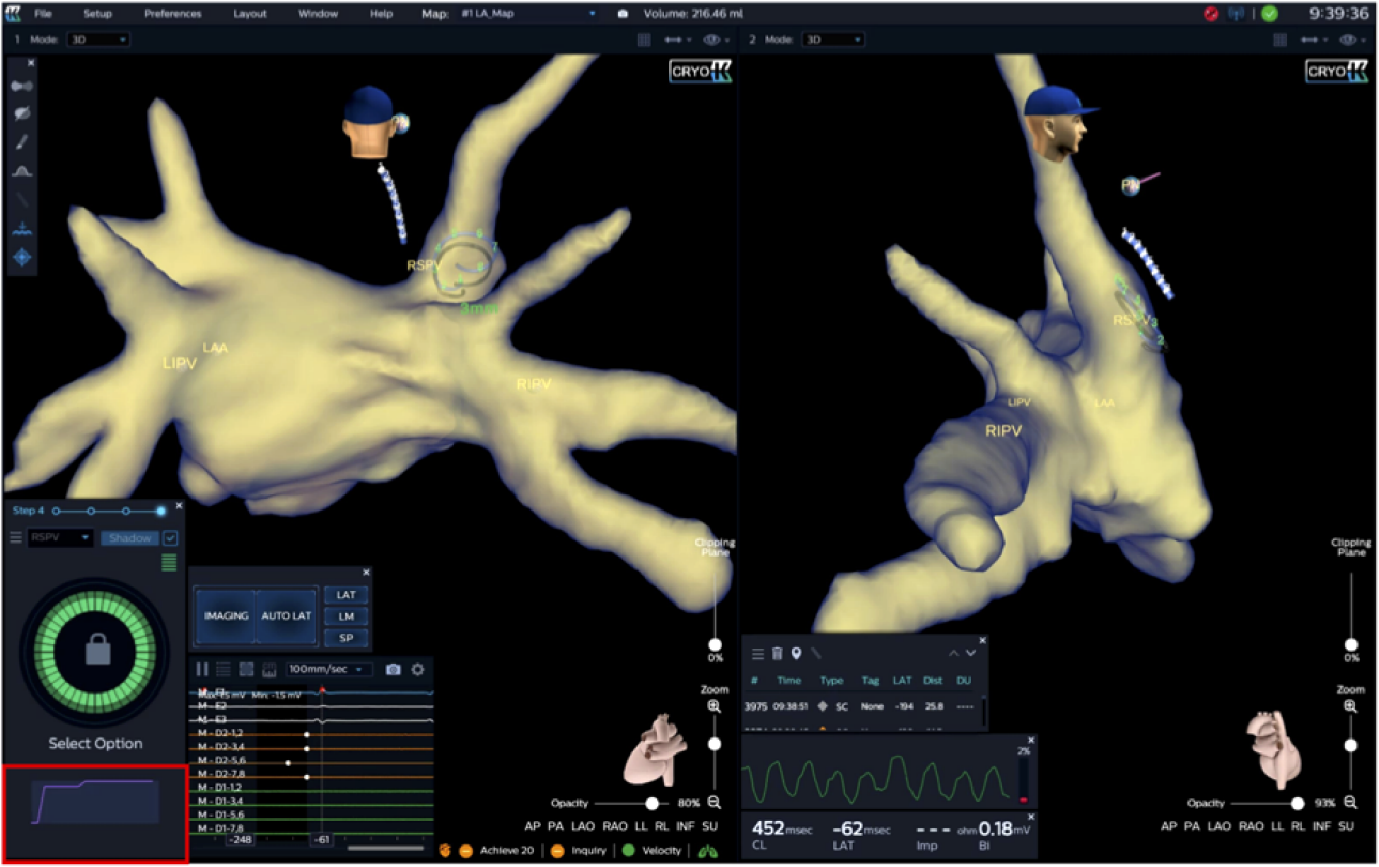
Immediately following injection, peak amplitude and rate of decay (corresponding to saline washout rate) over 10–15 s and final level are measured at the level of all Achieve electrodes. A slow decay to a novel baseline, as shown by the Waveform Graph (outlined in red) in the example below, displays total PV-occlusion, whereas a fast amplitude decay would indicate a leak (not shown). Furthermore, occlusion quality of the target PV is color-coded and, in this case, shows complete occlusion of the RSPV (green circle). Achieve catheter positions in right superior pulmonary vein (RSPV). RIPV, right inferior pulmonary vein; LIPV, left inferior pulmonary vein; LAA, left atrial appendage.

For 12 s post-injection, the system displays a representative dynamic waveform based on the mean voltages read off the Achieve electrodes. This informative waveform closely relates to the fluid's dissipation dynamics. After that, the system indicates either full occlusion or the presence of a residual PV to LA leak graphically (Figure 2). If there is a leak, blood will quickly pass over the electrodes and the electric field will return to the baseline value. The system displays a qualitative real-time waveform that allows the operator to assess whether there is PV occlusion or leak based upon the relative changes and the persistence or transience of the waveform.

Dye and saline differ substantially in their physical properties. Due to more favorable properties, especially with regard to conductivity, saline is potentially more sensitive in assessing leaks during PV-occlusion assessment as saline is more likely to reach all Achieve's electrodes.

**Figure 3:**
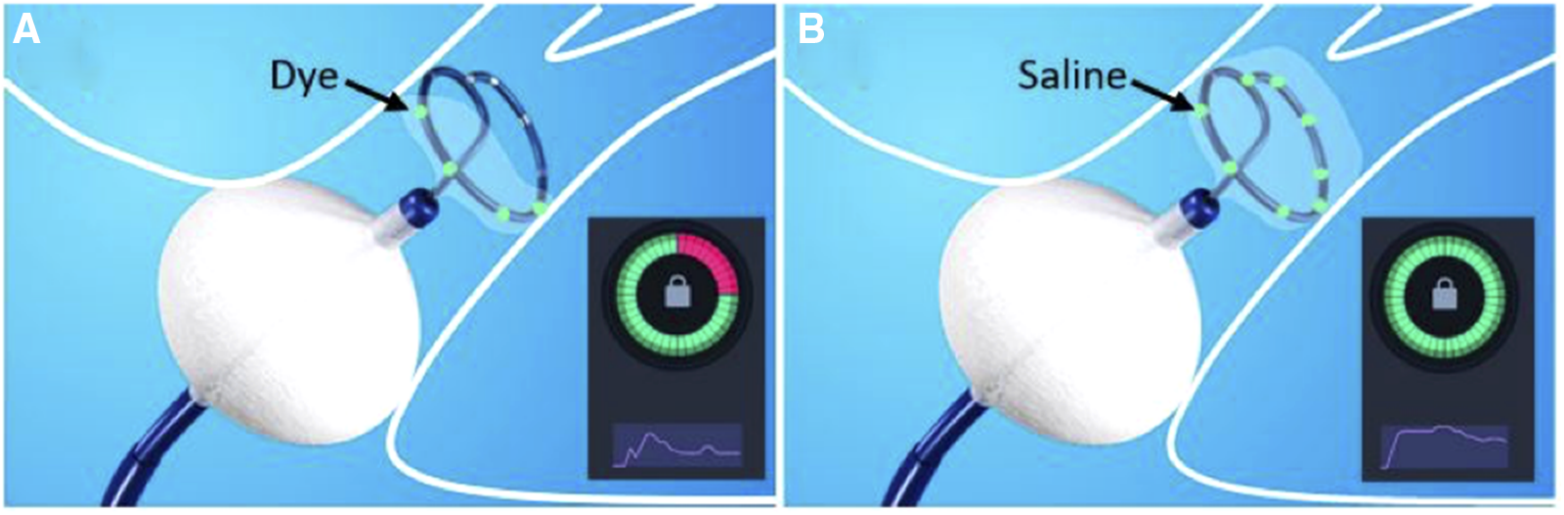
During the assessment of pulmonary vein (PV) occlusion with (**A**) dye and (**B**) saline injection, the inherent characteristic physical differences of the two fluids may impact the output of the PV occlusion evaluation. In this example, the injected dye is embedded and sensed by only four of the Archive’s electrodes (colored green, Figure 1A). In contrast, the injected saline has successfully embedded in all the electrodes (eight colored green, Figure 1B). Accordingly, dye injection has erroneously yielded a false-positive identification of a residual PV to LA leak (suboptimal partial PV occlusion, while the saline injection assessment has correctly indicated (true-positive) occlusion of the PV.

Figure 3 shows a representative example of occlusion testing (KODEX Occlusion Viewer) based on injection of dye (panel A) and saline (panel B) considering their different physical properties, illustrating how only four electrodes sense the injected dye, whereas all eight electrodes sense the Saline.

#### Periprocedural management

Prior to the procedure, transthoracic and transesophageal echocardiography was performed to rule out intracardiac thrombi and to assess the LA diameter and cardiac function.

AF ablation was performed on uninterrupted oral vitamin K-anticoagulation with an INR of 2.0–3.0 on the day of the procedure. In patients treated with novel oral anticoagulants (NOACs), anticoagulation was stopped the day before the procedure.

Pericardial effusion after ablation was ruled out in all patients by transthoracic echocardiography directly after the procedure and at the day of discharge.

Low molecular-weight heparin was administered in patients on vitamin K-antagonists with an International Normalized Ratio (INR) < 2.0 until a therapeutic INR of 2–3 was achieved. Pre-existing therapy with NOACs was reinitiated 6 h after the ablation procedure. Proton pump inhibitors were recommended for 6 weeks after ablation.

### Procedural management

Catheter ablation was performed under deep sedation. Vital parameters were continuously monitored. Heparin boluses were given targeting an activated clotting time >300 s. A diagnostic catheter was introduced *via* the right femoral vein and positioned within the coronary sinus. A temperature probe was placed within the esophagus at the level of the PVs to monitor esophageal temperatures during freeze cycles. The intraluminal esophageal temperature cut-off was set at 15°C. A single transseptal puncture was performed under fluoroscopic guidance using a modified Brockenbrough technique and a 8.5 F transseptal sheath (SL 0, St. Jude Medical, Inc, St Paul, MN) was introduced into the LA. The transseptal sheath was exchanged over a guidewire for a 15 F steerable sheath (Flexcath advance, Medtronic Inc).

In patients undergoing Cryo^EPD^ ablation a 3D anatomical image of the PVs was obtained using KODEX-EPD in a standardized sequence using a 15 mm or 20 mm circular mapping catheter (Achieve, Medtronic Inc, Minneapolis, US) and the steerable sheath. PV-occlusion was assessed by KODEX-EPD and its occlusion tool using 6–8 ml of 0.90% saline injection to assess PV occlusion. In patients of the Cryo group selective PV-angiographies were performed in order to determine PV anatomy. To verify total PV-occlusion by the CB, dye injection was used in these patients and occlusion was assessed *via* fluoroscopy.

In all patients the Achieve catheter was used to record real-time recordings from the targeted PV. In all patients, the fourth-generation CB (Arctic front advance pro, Medtronic Inc.) was used. A freeze-cycle duration of 180 or 240 s was applied after verification of a complete PV-occlusion. No empiric bonus freeze-cycle was added.

During ablation of the septal PVs, continuous phrenic nerve pacing at maximum output and pulse width (10 mA, 2.0 ms) at a cycle length of 750 ms was performed using a diagnostic catheter positioned in the superior vena cava. Phrenic nerve capture was monitored by tactile feedback of diaphragmatic contraction and registration of the compound motoric activation potential (CMAP) of the right diaphragm. Energy delivery was stopped immediately when diaphragmatic movement was weakened or lost or when the CMAP potential amplitude decreased by 30%.

### Data handling and statistical analyses

Data regarding patient demographics were collected retrospectively from patient medical records. Continuous data are given as mean ± standard deviation or median [1st quartile, 3rd quartile], and categorical variables are expressed as number and percentage.

The Pearson's *χ*^2^ test, Wilcoxon rank sum test or Fisher's exact test was used to compare both study groups with regard to procedural parameters. *P*-values <0.05 were considered significant. Statistical analyses were performed using R computing software 4.1.0.

## Results

### Patient population

A total of 140 patients, who underwent CB-based PVI, were analyzed. The study population comprised 48 (34%) females, 49 (35%) patients suffering from paroxysmal and 91 (65%) suffering from persistent AF. Mean patient age was 66 ± 12 years.

70 patients underwent CB-based AF ablation in conjunction with KODEX-EPD (Cryo^EPD^ group). 70 patients undergoing conventional CB-PVI acted as controls (Cryo group).

Detailed patient characteristics are summarized in Table 1.

**Table 1:** Baseline characteristics.

	Cryo^EPD^ group (*n* = 70)^a^	Cryo group (*n* = 70)^a^	*p*-value^b^
Age (years)	64 ± 13	68 ± 10	0.13
Female (*n*, %)	21 (30)	27 (39)	0.3
BMI (kg/m^2^)	27 ± 5	27 ± 6	0.8
Paroxysmal AF (*n*, %)	27 (39)	22 (31)	0.4
Persistent AF (*n*, %)	43 (61)	48 (69)	0.4
CHA_2_DS_2_-VASc score	2 [1; 3]	3 [2; 4]	**0.043**
Arterial hypertension (*n*, %)	42 (60)	53 (76)	**0.047**
Diabetes mellitus (*n*, %)	4 (6)	7 (10)	0.3
Heart failure (*n*, %)	19 (27)	22 (31)	0.6
Chronic kidney disease (*n*, %)	7 (10)	5 (7)	0.5
COPD (*n*, %)	3 (4)	5 (7)	0.7
OSAS (*n*, %)	4 (6)	6 (9)	0.5
Medical history of stroke and/or TIA (*n*, %)	5 (7)	8 (11)	0.4

AF, atrial fibrillation; COPD, chronic obstructive pulmonary disease; OSAS, obstructive sleep apnea syndrome; TIA, transient ischemic attack.

^a^ Values are mean ± SD, median [1st quartile, 3rd quartile] or *n* (%).

^b^ Wilcoxon rank sum test; Pearson’s *χ*^2^ test; Fisher’s exact test.

### Ablation characteristics and procedural parameters

In 140 patients a total of 560 PVs were identified. No left or right common PV was documented. All targeted PVs were successfully isolated, either verified by registration of “time-to-isolation” (TTI) or absence of PV potentials after ablation as assessed with the Achieve multipolar catheter.

The overall median number of CB applications per vein was 1 [1; 1] for the Cryo^EPD^ group and 1 [1; 1] for the Cryo group (*p* = 0.5). Overall total freeze duration was 240 [240; 240] seconds for the Cryo^EPD^ group and 240 [210; 240] seconds for the Cryo group (*p* = 0.12). A mean overall minimal temperature of −49 ± 7°C for the Cryo^EPD^ group and −48 ± 6°C for the Cryo group were observed (*p* = 0.4).

Mean total procedure time was 66 ± 15 min for the Cryo^EPD^ group, and 65 ± 19 min for the Cryo group (*p* = 0.3). Fluoroscopy time and dose area product were significantly reduced in the Cryo^EPD^ group when compared to the Cryo group (6 ± 4 min and 193 [111; 297] cGycm^2^ vs. 13 ± 6 min and 381 [268; 614] cGycm^2^, *p* < 0.001 and *p* < 0.001). 53 ± 18 ml contrast was used for the Cryo group, whereas no contrast was needed for the Cryo^EPD^ group (*p* < 0.001). TTI could be documented in 99/280 (35%) and 147/280 (52%) of all targeted PVs for the Cryo^EPD^ group and the Cryo group (*p* < 0.001). Mean mapping time to assess a real-time image of the LA and PVs during Cryo^EPD^ procedures was 5 ± 2 min.

Details on ablation and procedural parameters are given in Table 2.

**Table 2:** Detailed procedural characteristics.

	Cryo^EPD^ group (n = 70)^a^	Cryo group (n = 70)^a^	*p*-value^b^
Procedure time (min)	66 ± 15	65 ± 19	0.3
Fluoroscopy time (min)	6 ± 4	13 ± 6	**<0.001**
Dose area product (cGycm^2^)	193 [111; 297]	381 [268; 614]	**<0.001**
Total mapping time (min)	5 ± 2	/	
Volume contrast medium (ml)	0 ± 0	53 ± 18	**<0.001**
Median freeze cycles (*n*)			
Total	1 [1; 1]	1 [1; 1]	0.5
LSPV	1 [1; 1]	1 [1; 1]	0.5
LIPV	1 [1; 1]	1 [1; 1]	0.2
RIPV	1 [1; 1]	1 [1; 1]	0.09
RSPV	1 [1; 1]	1 [1; 1]	0.4
Median freeze duration (sec)			
Total	240 [240; 240]	240 [210; 240]	0.12
LSPV	240 [240; 240]	240 [210; 240]	0.12
LIPV	240 [240; 240]	240 [183; 240]	0.5
RIPV	240 [240; 240]	240 [180; 240]	0.3
RSPV	240 [240; 240]	240 [180; 240]	0.08
Minimum freeze temperature (°C)			
Total	−49 ± 7	−48 ± 6	0.4
LSPV	−49 ± 7	−48 ± 6	0.4
LIPV	−46 ± 6	−45 ± 6	0.4
RIPV	−50 ± 7	−48 ± 6	0.03
RSPV	−51 ± 6	−50 ± 6	0.2

^a^ Values are mean ± SD, median [1st quartile, 3rd quartile] or *n* (%).

^b^ Wilcoxon rank sum test.

#### PV occlusion assessment *via* occlusion tool using saline injection

A total of 1 [1; 1], 1 [1; 2], 2 [2; 3] and 1 [1; 2] occlusion checks using KODEX-EPD and its occlusion tool were needed for the LSPV, LIPV, RIPV and RSPV to confirm total PV occlusion. After initiation of a freeze cycle, freezing was stopped due to inadequate temperature drop in a total of 17 freeze cycles, whereby this was the case mostly during freezing of the RIPV (*n* = 6).

#### Periprocedural complications

There was no significant difference regarding overall complication rate between both groups (4/70 (5.7%) for the Cryo^EPD^ group vs. 6/70 (8.6%) for the Cryo group, *p* = 0.5).

Major complications occurred in 3/70 (4.3%; *n* = 3 phrenic nerve paralysis) patients of the Cryo^EPD^ group and in 5/70 (7.1%; *n* = 4 phrenic nerve paralysis, *n* = 1 cardiac tamponade) patients of the Cryo group (*p* = 0.7).

After a total follow-up duration of 85 ± 12 days 6/7 (86%) PNPs were still present, but asymptomatic.

## Discussion

This study reports on acute efficacy, fluoroscopy exposure and amount of dye applied during CB-based PVI in combination with KODEX-EPD and the saline injection workflow as compared to conventional CB-based PVI.

The major findings of the underlying study are as follows:

 1. KODEX-EPD guided CB-based AF ablation enables effective, dye-free CB-PVI in an all-comer patient cohort.
 2. KODEX-EPD guided CB ablation was associated with significantly reduced fluoroscopy times and dose areas products as compared to conventional CB ablation.
 3. Real-time electroanatomic mapping with a mean mapping time of 5 ± 2 min enables visualization of PV-anatomy and catheter navigation within the LA without compromising overall procedure duration.

Early Rhythm control in patients with AF has been shown to be beneficial either with AADs or catheter ablation (2). This has been demonstrated for all patient groups, and patients with higher comorbidity burden appear to benefit most from early rhythm control (3). In this context, early catheter ablation with save ablation tools preferentially single shot devices such as the CB are gaining more and more attention and the increasing importance of the CB, as an effective, safe and reproducible ablation tool for first-do PVI has recently been demonstrated (7, 8). However, the benefits of the currently established, conventional CB-based PVI are still restricted by relevant fluoroscopy exposures and necessity of for dye. Fluoroscopy is associated with potential harm for both, operators and patients. This includes delayed effects of radiation exposure with acute and subacute skin injury as well as radiation-induced cancer and genetic abnormalities (13). In addition, dye is commonly used to visualize the PVs and to assess PV occlusion prior to cryo-application. As of common knowledge, dye injection is associated with potential side effects such as allergic reactions or impairment of renal function (14). Alternative imaging modalities such as intracardiac or transesophageal echocardiography, have not prevailed due to several limitations such as a long learning curve, limited availability and additional cost (15, 16). Even after optimization of several procedural steps during CB ablation (17), fluoroscopy times remain substantial with 12–20 min reported in international multicenter trials (7, 8, 18). In this context fluoroscopy exposure of this cohort treated with the saline-injection based occlusion tool are remarkably low, not only when compared to the comparator group, but also when compared to the current literature. Two main aspects might play a major role for reduction of fluoroscopy times, namely the occlusion tool and its saline injection workflow and additional imaging with the KODEX-EPD which provides enhanced LA and PV visualization (9, 19) and facilitates catheter navigation and guiding. Of note, real-time electroanatomic mapping with a mean mapping time of 5 ± 2 min in this patient population enabled visualization of PV-anatomy and catheter navigation within the LA without compromising overall procedure duration (Table 2).

Acute efficacy of KODEX-EPD guided cryoablation has been demonstrated recently, however treatments were based on previous software versions where PV occlusion was assessed *via* the KODEX-EPD occlusion tool but still depending on dye injection (12, 19). Since introduction of the saline-based occlusion assessment, only one smaller study evaluated KODEX-EPD's novel saline injection workflow in a small cohort of 38 patients with promising results (20). However, no control group was included in this analysis and contrast was used in a certain number of patients to verify the PV occlusion assessment.

Thus, this is the first study to report on KODEX-EPD guided CB ablation procedures using the novel saline injection workflow in comparison to conventional CB-based PVI. It was demonstrated, that a KODEX-EPD guided CB-based AF ablation enables fast and effective, dye-free CB-PVI with significantly reduced fluoroscopy exposure in an all-comer patient cohort. The benefits of the novel system are of potential interest with regard to safety not only for patients but also for operators as for interventional electrophysiologists with high work intensity, a longterm total fluoroscopy exposure can lead to significant cumulative doses, and thus to an increased longterm risk [e.g., DNA damage, malignancy induction, (13)]. Furthermore, implementation of KODEX-EPD into the cryo-workflow and not having to use dye, might prevent allergic reactions and renal failure, and therefore might be especially favourable for a selected patient cohort (e.g., patients with known allergy or patients with severe renal insufficiency).

Besides procedure and fluoroscopy times, periprocedural efficacy is of importance. Compared to published data, the rate of documented TTI was low in our study, especially within the Cryo^EPD^ group. However, the focus of our study was to assess the feasibility of CB ablation in combination with KODEX-EPD, and the reliability of the occlusion tool using saline injection and a comparison to conventional cryoablation with regard to fluoroscopy and dye usage. Therefore, the TTI was not focused on. But in both patient groups of this study acute PV isolation rates were comparable. Furthermore, number of cryo-applications per PV as well as nadir freezing temperatures were comparable, underlining comparable acute efficacy.

### Strengths and limitations

This is the first study to report on KODEX-EPD guided CB ablation procedures using the novel saline injection workflow in comparison to conventional CB-based PVI within a large all-comer patient cohort. The main goal of this study was to assess the feasibility of cryoablation in combination with KODEX-EPD, the reliability of the occlusion tool using saline injection and a comparison to conventional cryoablation with regard to fluoroscopy and dye usage. However, as this is a retrospective non-randomized trial patients demographic slightly differ between both groups with regard to CHA_2_DS_2_-VASc score and incidence of arterial hypertension. Furthermore, due to the novelty of the system, we cannot provide follow-up data with regard to long-term arrhythmia-free survival.

Further multi-center and randomized studies are desirable to confirm our findings.

## Conclusion

This study demonstrates, that KODEX-EPD guided CB-based AF ablation enables dye-free PVI with reduced fluoroscopy exposure when compared to conventional CB-PVI, while maintaining accustomed procedure duration, acute efficacy and periprocedural safety.

## Data Availability

The raw data supporting the conclusions of this article will be made available by the authors, without undue reservation.
